# Assessment of the Impact of a Short-Term Intervention on Menstrual Hygiene Practices of Adolescent Girls in Rural Parts of Central India

**DOI:** 10.7759/cureus.44933

**Published:** 2023-09-08

**Authors:** Keta Vagha, Shraddha Sawhney, Ashish Varma, Jayant D Vagha, Naman Mishra

**Affiliations:** 1 Department of Pediatrics, Jawaharlal Nehru Medical College, Datta Meghe Institute of Higher Education and Research, Wardha, IND

**Keywords:** health education, adolescents, infection, sanitary pads, menstruation

## Abstract

Introduction

Menstruation affects many young girls in a negative way in relation to their physical and psychological health despite being a reasonably common issue in daily life. Their health relies on their learning about fundamental menstruation hygiene. They have a significant risk of developing ailments due to their lack of education in this area. Young girls can avoid several preventable illnesses by being educated about menstruation hygiene and practices. Therefore, our study aimed to assess the impact of a health education intervention on the knowledge, attitude, and practice of teenage girls about menstrual hygiene.

Materials and methods

The current educational interventional study design evaluates the impact of an educational intervention on menstrual hygiene in teenage females. Adolescent girls were educated about the practices and taboos related to menstruation. They were assessed using a pretest and post-test after the intervention. The results of the pre- and post-study tests were compared, and the effect of the intervention was determined.

Result

A noteworthy observation of the study was that only 87 (34.8%) teenage girls were aware of hygienic practices before menarche. The didactic lecture had a favorable impact, and after two months, 2.40% of the 6.80% of females who changed their reusable pads just once a week began replacing them every two days. Additionally, a shift in hygiene behaviors was noted; 17.20% of the 33.60% of individuals started washing their hands with soap and water after changing their menstruation pads, which was significant (p=0.05).

Conclusion

The study assessed the impact of a short-term intervention on the menstrual habits practiced by adolescent girls. We concluded that an intervention in the form of educational counseling can significantly impact the menstrual hygiene practices of adolescent girls. Teaching teenage females resulted in a considerable improvement in their menstruation habits, which has a beneficial overall effect.

## Introduction

Adolescence is a period in life where an individual goes through a shift between childhood and adulthood. It is drastically influenced by the physical, psychological, and social changes around them. The beginning of menstruation in teenage girls is the most notable change. Menstruation is a regular biological process that involves the periodic ejection of blood and mucosal tissue from the uterus [1]. Adolescence is a very vulnerable phase in a girl’s life. With the development of secondary sexual characteristics and hormonal changes, they are at a greater risk of diseases and social exploitation. In practically every community in the world, menstruation is associated with a cultural stigma. Despite being a relatively frequent issue in everyday life, menstruation is related to numerous unpleasant attitudes among young girls [2]. Menstruating females have been socially prohibited, culturally restricted, and physically isolated, reinforcing a mainstream paradigm [3]. This attached stigma has further led to a lack of knowledge and conversations about menstrual hygiene. Adolescent females who are unaware of their menstrual cycle face negative cultural attitudes and participate in unhygienic menstruation practices [4]. Many teenage girls do not practice appropriate menstrual hygiene at home, at school, or in other public situations because of a combination of judgemental social circumstances, misinformation, inadequate facilities, and a shortage of absorbent material options.

The knowledge and wellness of teenage girls depend on their learning about basic menstrual hygiene. Females are often unprepared, particularly in rural areas, in terms of menstrual cycle information, behaviors, and attitudes [5]. According to several studies, there is a significant disparity in menstruation hygiene between teenage girls in rural and urban areas [2]. This knowledge gap is often the main reason that leads to increased infections and diseases in the former. More people are becoming aware of the negative effects that unmet menstrual health needs have on the physical, mental, and social well-being of girls, which has prompted policy and program responses globally [6]. The Government of India has proposed menstrual hygiene schemes under the national health mission that aim to educate adolescent girls in the age group of 10-19 years and has incentivized Accredited Social Health Activist (ASHA) workers to provide subsidized sanitary pads under the scheme [7]. Poor hygiene practices predispose to genital and urinary tract infections. The socioeconomic factor plays a significant role when it comes to maintaining safe and hygienic menstrual practices. Hygiene measures during menstruation are critical since they raise the risk of psychological, physiological, and medical complications such as skin abrasion and reproductive tract infections [3,8]. According to studies, most adolescent females have minimal and poor knowledge of menarche and menstruation [4]. Therefore, our study aims to determine how health education interventions have an impact on the menstrual hygiene practices of teenage girls.

## Materials and methods

Design study

This study was an educational interventional study. It was conducted in two phases: the first phase assessed the knowledge, attitude, and practices of menstrual hygiene, and the second phase was an educational lecture that provided an understanding of the health hazards poor menstrual hygiene practices posed on girls.

Study area

This prospective interventional study was conducted by researchers from the Department of Pediatrics, Acharya Vinoba Bhave Rural Hospital, Sawangi (Meghe) located in Maharashtra, India, at three different rural schools in the area.

Sample size and study population

The research comprised adolescent girls from three different schools, and the total number of participants added up to 250. The sample size was calculated considering a 95% confidence interval and 5% margin of error. The total population of students across the three schools in grades 8-10 was 710, of which the population proportion of girls was 50%, making the sample size 250. The schools were randomly selected, and students from grades 8-10 who had attained menarche were included in the study. The response rate was 98%.

Intervention

The study was initiated after obtaining permission from the Institutional Ethics Committee of Datta Meghe Institute of Medical Science (Deemed to be University) (approval number: DMIMS(DU)/IEC/2022/433). After obtaining approval from the school administration of Indira High School, the objective of the research was conveyed to the girls in grades 8-10 who had attained menarche, and verbal consent was acquired from them and their parents. After explaining to the girls the predesigned questionnaire in detail, they were requested to complete it [9,10]. This structured questionnaire covered issues such as menstruation awareness, sources of knowledge about menstruation, menstrual hygiene, and restricted activities during this period. The participants were then educated and counseled on the ill effects of poor menstrual hygiene via a didactic lecture. The schools were revisited after two months, and the questionnaire was again given to the girls to assess the outcome of the short-term intervention in the form of a didactic lecture done during the last visit. After obtaining the data from both visits, the results of the pre- and post-study tests were compared to assess the impact of the intervention. Since the questions involved both themes including knowledge and practices such as willingness to attend school and participate in sports, the impact was ascertained.

Data analysis

Data were processed using statistical software tools to deliver results and inferences. Data analysis was done using Statistical Package for the Social Sciences (SPSS) version 25.0 (IBM SPSS Statistics, Armonk, NY, USA) and Microsoft Excel 2019 (Microsoft Corporation, Redmond, WA, USA). The analysis was carried out between the pre- and post-study answers using the McNemar test. P-values were calculated, and those less than 0.05 were considered significant.

## Results

The study involved adolescent girls belonging to age groups 13-16 years who had attained their menarche. A total of 250 students were considered, and their consent was obtained for the study. Of the 250 adolescent girls from different rural schools in central India, 46 (18.4%) were 13 years old, 11 (4.4%) were 14 years old, 105 (42%) were 15 years old, and 88 (35.2%) were 16 years old. The students belonged to grades 7, 8, and 9. Forty-eight (19.20%) students were in the seventh grade, 84 (33.60%) were in the eighth grade, and 118 (47.20%) were in the ninth grade (Table 1).

**Table 1:** Sociodemographic characteristics of the adolescent girls (N=250)

Age (years)	Number	%
13	46	18.40
14	11	4.40
15	105	42
16	88	35.20
Grade	Number	%
7	48	19.20
8	84	33.60
9	118	47.20

Among the types of pads used, 97 (38.80%) girls gave information about the use of cotton pads, 65 (26%) girls used reusable pads, 32 (64%) girls reported the use of both cotton and cloth combined, and 56 (22.40%) girls used only cloth while they were menstruating. An important observation made in the study was that 163 (65.20%) adolescent girls had no knowledge about menstrual and hygiene practices before the attainment of menarche, while only 87 (34.80%) girls were aware of hygienic practices before they had attained menarche. These girls were then asked about the source of their information regarding hygiene practices, of which 131 (52.40%) girls gained this information from their mothers or guardians, 91 (36.40%) girls were made aware of these practices by their friends, and 28 (11.2%) girls were educated by the media. To understand the thoughts and beliefs of these young girls regarding menstruation, they were asked if they believed that menstruation was a disease that they suffered from or a naturally occurring phenomenon. The result obtained showed that 224 (89.6%) girls thought it to be a naturally occurring phenomenon, while 26 (10.4%) thought of it as a disease.

The frequency of changing reusable menstrual pads that they used while menstruating when they were outside of their home or in school was asked, and 17 (6.80%) girls gave the history of changing their pads once per week, 137 (54.8%) girls reported changing the products in a two-day interval, 64 (25.6%) girls reported changing the soaked products every day, and 32 (12.80%) girls changed them twice in a day. Following the change of the soaked products while outside the comfort of their homes, the girls were questioned about the frequency of changing the used product in a day(24 hours). A total of 107 (42.80%) girls reported changing their pads more than three times a day, 69 (27.60%) girls used to change it three times a day, 52 (20.80%) girls changed it two times, and 22 (8.80%) girls used only one pad throughout the day.

The girls were further asked about their maintenance of hand hygiene following the change of soaked menstrual products, and 166 (66.40%) girls used to clean their hands following the change of the menstrual products, while 84 (33.60%) girls did not wash their hands. Only 82 (26.90%) girls adhered to the hygiene practice of cleansing their external genitalia each time they changed their pads. Only 107 females, or 38.97% of the total, washed their external genitalia with soap and water.

The important aspect of dumping the used menstrual waste was enquired about as it is an open source of infection if not disposed of properly. Fifty-seven (22.80%) girls disposed of their used products in the toilet by flushing it, 119 (47.60%) used to burn the used pads as they were told by their family, 22 (8.80%) disposed of the pads in the municipal dustbin, 36 (14.40%) buried it in a nearby field, and 16 (6.4%) were unaware about the harmful effect and used to wash the pad and reuse it (Table 2).

**Table 2:** Knowledge about menstruation and practices followed by the adolescent girls

Variable	Number	%
Types of pads used		
Cotton pads (sanitary pads)	97	38.80
Reusable pads	65	26
Cloth	56	22.40
Both cotton and reusable pads	32	12.80
Knowledge about menstruation before attaining menarche		
Girls who knew	87	34.80
Did not know	163	65.20
Frequency of changing reusable pads		
Once a week	17	6.80
Two-day interval	137	54.80
Every day	64	25.60
Twice in one day	32	12.80
Frequency of changing pads in a day (24 hours)		
More than three times	107	42.80
Three times	69	27.60
Two times	52	20.80
Once	22	8.80
Hand wash after changing pads		
Washed hands	166	66.40
Did not wash hands	84	33.60
Method of disposal		
Flush	57	22.80
Burn	119	47.60
Dustbin	22	8.80
Bury	36	14.40
Wash and reuse	16	6.40

The method of washing the cloth or the pad that they reused was also asked to understand whether it was cleaned properly with soap and water or it was just rinsed with water. Sixty-eight (27.20%) girls just rinsed or washed it with water, and 182 ( 72.80%) girls used soap to wash it before reusing it. Since the pad, if disposed of without a covering, can serve as a source of infection, the girls were asked whether they wrapped the soiled pads before disposing of them. Fifty-three (21.20%) girls disposed of them uncovered, while 197 (78.80%) girls wrapped them with double-layered paper before disposing.

A total of 206 (82.40%) girls gave the history of using the same spot to urinate at home during their menses as they used while they were not menstruating, while 44 (17.60%) girls were not allowed to use the same spot to urinate while menstruating that they used normally. Regarding the opinion of these girls on playing, dancing, or any physical activity that was considered harmful while menstruating, 171 (68.4%) girls thought that it was harmful, while (79 31.6%) girls were actively indulging in physical activity while menstruating. Seventy-nine (31.6%) girls out of the total avoided going to school and preferred to stay at home.

After the intervention, which was in the form of a didactic lecture, the adolescent girls were again questioned about their menstrual practices after a duration of two months. The didactic lecture showed positive effects, and the results obtained after two months of intervention are as follows. Six (2.40%) out of the 17 (6.80%) girls who changed their reusable pads only once a week started changing them at two-day intervals. Out of the 22 (8.80%) adolescent girls who used a single pad throughout the day, eight (3.20%) started changing their pads two to three times a day. There was also a change observed in hygiene practices, as 43 (17.20%) of the 84 (33.60%) girls started washing their hands with soap and water after changing their menstrual pads (p<0.05), which was significant. The questionnaire revealed that of the 53 (21.20%) girls who were disposing of their pads uncovered previously, 25 (10%) changed this habit to wrapping it properly before disposal; the result showed a statistically significant difference after the intervention. Of the 171 girls who initially believed that doing any physical activity such as playing and dancing was harmful, 62 now actively took part in moderate activities, which was also a significant shift. Of the 79 girls who withdrew from school during their cycles, 32 resumed going to school after the intervention. This change was also significant (Table 3).

**Table 3:** Post-intervention comparison of practices NS: nonsignificant, S: significant

Variable	At the start of the study		After two months		McNemar test value	P-value
	Number	%	Number	%		
Frequency of change of reusable pads once a week	17	6.80	11	2.40	1.36	0.24 (NS)
Changed pad only once a day	22	8.80	14	3.20	1.91	0.16 (NS)
Did not wash hands after changing pad	84	33.60	41	17.20	19.72	0.0001 (S)
Uncovered disposal of pads	53	21.20	28	10.00	9.20	0.0021 (S)
Thought physical activity is harmful	171	68.40	109	43.60	31.20	0.0001 (S)
Avoided going to school during cycle	79	31.60	47	18.80	10.86	0.0010 (S)

The study shows that educating adolescent girls produces a significant change in their menstrual practices, leading to an overall positive impact. While changes in the menstrual and hygiene practices were observed as a result of the educational lecture, no changes were observed in the practices followed at home, such as using different washrooms to urinate during menstruation and avoiding going to religious places and the kitchen during the cycle.

## Discussion

The life of a girl during her adolescence is one of great vulnerability. They are more vulnerable to illnesses and social exploitation because of hormonal changes and the development of secondary sexual traits. Menstruation carries a social stigma in almost every community in the world. It affects young girls in a variety of negative ways, despite being a reasonably common issue in daily life. Further resulting from this associated stigma is a lack of information and knowledge regarding menstrual hygiene. Teenage girls who are ignorant of their period have negative cultural views and engage in unsanitary menstrual habits. Therefore, this prospective interventional study was not only taken up to assess the knowledge, attitude, and practices but also to educate them about menstrual hygiene and its ill effects if not maintained. A total of 250 adolescent girls belonging to the age group of 13-16 years who attained their menarche were included in the study and were provided with a questionnaire assessing their knowledge, attitude, and practices regarding menstruation. These girls were educated regarding proper menstrual hygiene practices via a didactic lecture, and their knowledge, attitude, and practices were reassessed after two months.

Boakye-Yiadom et al. [11] conducted a similar study in Ghana where they divided their population into two groups: girls aged 10-14 years and those aged 15-19 years. They found that there was almost an equal number of girls in the study belonging to both groups, 203 and 209, respectively. We, on the other hand, divided the population according to the different ages in the adolescent age groups; therefore, of the 250 adolescent girls included in our study from three different rural schools in central India, 18.4% were 13 years old, 4.4% were 14 years old, 42% were 15 years old, and 35.2% were 16 years old. A similar study conducted by Bhatt et al. [12] revealed that 8.14% of 86 girls used cloth as the absorbent material during menstruation, and 91.86% of girls used market-bought sanitary napkins. Another study by Pramodha et al. [13] conducted in the rural Kannada region showed that 63.85% of adolescent girls still used cloth, whereas 36.15% used sanitary napkins. In our study, 38.80% of the girls used sanitary napkins, 26% used reusable pads, 12.80% used both cotton and cloth, and 22.40% used cloth only. Yadav et al. [14] conducted a cross-sectional study at 11 different schools from seven villages in Nepal with a sample size of 276 girls. They had an extensive questionnaire that included questions on physiology and beliefs about menstruation, in which they found that 67.4% of girls had fair knowledge, 26.4% had good knowledge, and 6.2% had poor knowledge. Our study had similar results in the knowledge aspect, where we found that 64.8% had fair knowledge, 28% had good knowledge, and 7.2% had poor knowledge. In the study conducted by Bhatt et al. [12], they determined the various sources of acquiring knowledge regarding menstruation in their study population, which showed most of the girls getting this knowledge from their mothers, followed by teachers, and then social media, such as the internet or television; these results are consistent throughout the studies as it showed a similar trend. A prospective observational study conducted by Rajavardhana et al. [15] deduced that 88% of the study population used sanitary napkins available in the market, 5.6% used cloth, and 3.2% used both products, whereas the remaining 3.2% used other products such as tampons. These results are contrary to our findings, where we found that 38.8% used market-available sanitary napkins, 22.4% used cloth, 26% used reusable pads, and 12.80% used both cotton and reusable pads, showing poorer menstrual practices in our region.

The current study found that 38.8% of girls used sanitary pads during their periods, which is comparable to studies conducted in Madhya Pradesh in 2021 by Bali et al. [16], which showed the usage as 31%, and in Dakshina Kannada in 2021 by Pramodha et al. [13], which found that 36.15% of teenage girls used sanitary pads during their periods. Contrarily, studies done in Karnataka in 2020, Andhra Pradesh in 2016, and Uttarakhand in 2021 revealed that the utilization was, 70%, 78.5%, and 79.5%, respectively, while a study in Jodhpur, Rajasthan, in 2020 revealed that 85% of adolescent girls used sanitary pads throughout their menstrual cycle [17].

In our study, 66.4% of girls adhered to the standards of menstrual hygiene, which is comparable to the study by Thakre et al. [8] in Nagpur, where 58.09% of girls did the same. This is greater than the percentage found in the study by Patavegar et al. [18] in Delhi, in which only 34.32% of girls adhered to the standards. Shoor reported that 76% of women who had menstruation changed their sanitary napkins or cloth between two and three times each day, 16.3% four times per day, and 7.7% twice each day [19]. These results were not comparable to our study, which indicated that 42.80% of women changed their menstruation pads more than three times per day, 27.6% changed them three times per day, 20.80% changed them twice, and 8.80% changed their pads just once each day. One possible explanation for this discrepancy is the financial stability of adolescent females in rural and urban environments.

Limitations

The results obtained in this study were based on the information provided by the students as a part of the answers to the questionnaire and were not validated physically. Regular checkups and a check on their menstrual practices were not made.

## Conclusions

Poor menstrual hygiene is one of the leading causes of disease and infection in adolescent girls. We can assess the gravity of poor menstrual hygiene practices in rural India by finding its knowledge, attitude, and practices and finding out a few responsible factors causing this. We can also suggest some steps for society and administration as preventive measures to curb the spread of diseases due to improper hygiene in the vulnerable population of adolescents. We can gain knowledge about factors that are helpful in restoring their health back to normal. An hour of counseling and education about awareness of poor hygiene practices can make an impact on budding brains, and consideration of such sessions in the curriculum can be advocated. This will lead to a reduced prevalence of reproductive tract infections associated with poor menstrual hygiene and improve the lifestyle of the upcoming generation.

## Appendices

The questionnaire that was used in this study that covered issues such as menstruation awareness, sources of knowledge about menstruation, menstrual hygiene, and restricted activities during this period is shown in Table 4.

**Table 4:** Study questionnaire

Questions	Answer options				
Did you know about menstruation and its hygiene practices before attaining menarche?	Yes		No		
What do you think menstruation is?	Disease		Natural process		
Type of pads used	Cotton	Cloth/reusable pads		Both	
How many times a day do you change your pads during your last period (24 hours)?	Everyday	Twice in one day	Two days	Once a week	
Do you wash your hands before and after you change your pads?	Once	Twice	Thrice	More than three times	
Do you wash your genitals at the time of menstruation?	Yes		No		
Where do you dispose of the menstrual material after use?	In the toilet	Burn	Dustbin	Wash and reuse	Bury
Whom did you learn about menstruation?	School	Mother		Friend	
Do you usually wrap your used sanitary products before throwing them away?	Yes		No		
How do you wash the reusable cloth or pad?	With water		With soap and water		
Do you utilize the same spot to urinate at home during your last menstrual cycle as you do when you don’t have your period?	Yes		No		
Do you avoid going to certain places when you are menstruating, like the kitchen?	Yes		No		
Do you think it is harmful to dance or play while menstruating?	Yes		No		
Do you avoid going to school while you are menstruating?	Yes		No		
Why do you avoid going to school while menstruating?	Due to fear of leakage	Due to pain	No suitable place for disposable	Other reason	
